# Cardiometabolic Risk Factors and Adverse Outcomes in Pregnant Women With Type 2 Diabetes

**DOI:** 10.1210/jendso/bvaf146

**Published:** 2025-09-09

**Authors:** Anna Koefoed, Per Glud Ovesen, Jens Fuglsang, Astrid Fur, Dorte Møller Jensen, Lise Lotte Andersen, Emilie Rosbach, Peter Damm, Elisabeth Mathiesen, Anne Sørensen, Trine Tang Christensen, Harold David McIntyre, Sine Knorr, Ulla Kampmann

**Affiliations:** Department of Obstetrics and Gynecology, Aarhus University Hospital, DK-8200 Aarhus N, Denmark; Department of Clinical Medicine, Aarhus University, 8200 Aarhus N, Denmark; Steno Diabetes Center Aarhus, Aarhus University Hospital, 8200 Aarhus N, Denmark; Department of Obstetrics and Gynecology, Aarhus University Hospital, DK-8200 Aarhus N, Denmark; Department of Clinical Medicine, Aarhus University, 8200 Aarhus N, Denmark; Steno Diabetes Center Aarhus, Aarhus University Hospital, 8200 Aarhus N, Denmark; Department of Obstetrics and Gynecology, Aarhus University Hospital, DK-8200 Aarhus N, Denmark; Department of Clinical Medicine, Aarhus University, 8200 Aarhus N, Denmark; Steno Diabetes Center Aarhus, Aarhus University Hospital, 8200 Aarhus N, Denmark; Department of Clinical Medicine, Aarhus University, 8200 Aarhus N, Denmark; Steno Diabetes Center Odense, Odense University Hospital, 5000 Odense C, Denmark; Department of Obstetrics and Gynecology, Odense University Hospital, 5000 Odense C, Denmark; Department of Obstetrics and Gynecology, Odense University Hospital, 5000 Odense C, Denmark; Center for Pregnant Women with Diabetes, Department of Endocrinology and Obstetrics, Rigshospitalet, 2100 Copenhagen Ø, Denmark; Department of Clinical Medicine, University of Copenhagen, 2200 Copenhagen N, Denmark; Center for Pregnant Women with Diabetes, Department of Endocrinology and Obstetrics, Rigshospitalet, 2100 Copenhagen Ø, Denmark; Department of Clinical Medicine, University of Copenhagen, 2200 Copenhagen N, Denmark; Department of Obstetrics and Gynecology, Aalborg University Hospital, 9000 Aalborg, Denmark; Department of Clinical Medicine, Aalborg University, 9260 Gistrup, Denmark; Steno Diabetes Center Aalborg, Aalborg University Hospital, 9260 Gistrup, Denmark; Department of Obstetrics and Gynecology, Aarhus University Hospital, DK-8200 Aarhus N, Denmark; Mater Research, Faculty of Medicine, The University of Queensland, South Brisbane, QLD 4101, Australia; Department of Clinical Medicine, Aarhus University, 8200 Aarhus N, Denmark; Steno Diabetes Center Aarhus, Aarhus University Hospital, 8200 Aarhus N, Denmark; Department of Clinical Medicine, Aarhus University, 8200 Aarhus N, Denmark; Steno Diabetes Center Aarhus, Aarhus University Hospital, 8200 Aarhus N, Denmark

**Keywords:** diabetes in pregnancy, preexisting diabetes, pregnancy outcomes, risk factors, type 2 diabetes

## Abstract

**Context:**

The prevalence of type 2 diabetes (T2DM) in pregnancy is increasing rapidly worldwide. Consequently, there is a need to gain more knowledge about pregnant women with T2DM.

**Objective:**

The current study aimed to assess the prevalence of cardiometabolic risk factors and comorbidities in women with T2DM before and during pregnancy, and to evaluate associations with adverse obstetric and perinatal outcomes. A comparison between women with preexisting T2DM (P-T2DM) and women with T2DM first recognized during pregnancy (N-T2DM) was also performed.

**Design:**

A retrospective Danish national population-based cohort study, including all pregnancies in women with T2DM, giving birth to a live infant after 24 weeks of gestation, from 2004 until 2019.

**Results:**

The population included 1297 pregnancies in women with T2DM (1207 P-T2DM, 90 N-T2DM). Before pregnancy, 20.4% smoked, 13.4% had chronic hypertension, 12.5% had dyslipidemia, and 63.1% had obesity. Only 58.6% of women with P-T2DM entered pregnancy with hemoglobin A1c (HbA1c) < 53 mmol/mol (7.0%). During pregnancy, 73.4% had an excessive weight gain, and 21.6% attained a median HbA1c < 38 mmol/mol (5.6%). Preeclampsia was observed in 9.1% of women, preterm delivery in 19.0%, and large-for-gestational-age birthweight in 32.2% of infants. Obstetric and perinatal outcomes were similar in women with P-T2DM and N-T2DM. All modifiable exposures were associated with one or more severe adverse outcomes.

**Conclusion:**

Cardiometabolic risk factors are prevalent in pregnant women with T2DM and are associated with severe adverse outcomes for mother and child.

The prevalence of type 2 diabetes (T2DM) in pregnancy is increasing rapidly worldwide [1, 2]. As a result, there is an urgent need to improve the knowledge of pregnant women with T2DM.

Insulin resistance is a key factor in pregnancy [3] and as insulin resistance plays an important role in the pathophysiology of T2DM, it is expected to be worse in pregnant women with T2DM. In addition, pregnant women with T2DM are often older, have more obesity, and show a greater ethnic variation. Therefore, pregnant women with T2DM may encounter different challenges during pregnancy, compared to women with type 1 diabetes (T1DM), potentially contributing to a risk of treatment failure, hyperglycemia, and adverse outcomes.

Maternal hyperglycemia is associated with severe obstetric complications [1]. It generates an excessive nutrient supply to the fetus, associated with fetal adiposity and childhood metabolic dysfunction [4, 5]. Furthermore, maternal hyperglycemia is associated with congenital malformations, stillbirths and neonatal deaths in women with preexisting diabetes [6]. Therefore, achieving normoglycemia is crucial, emphasizing the importance of effective healthcare programs.

Apart from a recent study by Murphy et al [6], most studies on diabetes in pregnancy have included small numbers of women with T2DM [7-13]. Consequently, current guidelines for the management of preexisting diabetes during pregnancy are primarily based on studies on women with T1DM [14]. Furthermore, pregnant women with T1DM and T2DM are treated similarly [15], although they differ in fundamental etiology and pathogenesis of the disease and are managed differently outside pregnancy [16].

Concerningly, in the study by Murphy et al, the rate of perinatal deaths was higher in pregnancies complicated by T2DM compared to T1DM, associated with poor glycemic control [6]. This highlights the severity of the condition.

In the last decade, national guidelines have included the diagnosis “presumed preexisting diabetes first recognized during pregnancy.” As prepregnancy screening and treatment have not been provided for these women, they may be at a greater risk of experiencing adverse outcomes during pregnancy than women diagnosed with diabetes before conception. However, this has not been elucidated.

In this study we aimed to assess the prevalence of cardiometabolic risk factors in women with T2DM both before and during pregnancy, based on a large national Danish cohort, and to evaluate the association with adverse obstetric and perinatal outcomes. In addition, we wanted to compare women with preexisting T2DM (P-T2DM) and women with new T2DM (N-T2DM) regarding risk factors and adverse outcomes.

## Methods

### Study Design and Population

This retrospective Danish nationwide population-based cohort study included all identifiable pregnancies in women with T2DM, who were giving birth to a live infant after 24 weeks of gestation, from January 2004 until November 2019. Antenatal and perinatal care of all women with T2DM in Denmark was centralized at 4 hospitals. The study population was stratified into 2 groups: pregnancies in women with known preexisting T2DM (P-T2DM) and pregnancies in women with new T2DM first recognized during pregnancy and confirmed postpartum (N-T2DM). In Denmark, women are selectively screened for diabetes after becoming pregnant according to a set of risk factors: maternal prepregnancy body mass index (BMI) ≥27 kg/m^2^, family disposition to diabetes, previous birth of a child ≥4500 g, previous gestational diabetes, polycystic ovarian syndrome, multiple pregnancy, and glucosuria (discovered in a urine sample at any time during pregnancy).

Diagnostic criteria used during pregnancy were fasting venous plasma glucose level ≥7.0 mmol/L, random venous plasma glucose level ≥11.1 mmol/L, or hemoglobin A1c (HbA1c) ≥48 mmol/mol (6.5%) before 20 weeks of gestation [15]. Guidelines for treating and managing diabetes in pregnancy did not distinguish between preexisting and new diabetes [15].

### Data Sources and Collection

The study population was identified using the International Classification of Diseases Coding System 10th Edition (ICD-10). Diagnoses were validated during data collection from the medical records. All data included in the study were obtained from computerized hospital databases (electronic medical records and fetal ultrasound scan databases) or paper-based medical records in electronic or physical archives and typed into a study database.

### Exposure Variables

Population characteristics of interest were baseline variables (ethnicity, age and parity) and preexisting cardiometabolic risk factors (smoking, BMI), comorbidities including medical treatments, duration of diabetes, treatment of diabetes, and glycemic control). Furthermore, treatment of diabetes, glycemic control, and weight gain during pregnancy were also examined. Oral antidiabetic agents were not used during pregnancy.

Maternal age and duration of diabetes were calculated at the time of delivery. Prepregnancy body weight and height, ethnicity, and smoking were self-reported by the women and documented by the clinicians in the medical records. Antenatal data on maternal weight and daily insulin dose were collected at all visits to the outpatient clinics. Weight change during pregnancy was calculated as a difference in kg from prepregnancy weight. To compare with Danish guidelines, mean weight change during pregnancy was stratified according to the prepregnancy BMI [15]. Women with a BMI <25.0 kg/m^2^ were recommended to gain 10 to 15 kg, women with a BMI 25.0-29.9 kg/m^2^ 5 to 8 kg, and women with a BMI ≥30 kg/m^2^ 0 to 5 kg.

Prepregnancy HbA1c and HbA1c in the third trimester were categorized according to Danish guidelines [15]. Target HbA1c was <53 mmol/mol (7.0%) before pregnancy, <48 mmol/mol (6.5%) in early pregnancy, and <38 mmol/mol (5.6%) in late pregnancy. HbA1c measurements from 3 months before conception until delivery were included.

Data were collected on all preexisting chronic disorders using ICD-10 at the chapter level; short-term acute diseases were excluded. Prevalent comorbidities were grouped on 3- or 4-digit code levels. Treatment with statins, angiotensin-converting enzyme inhibitors, and angiotensin receptor blockers were considered exposure to potentially teratogenic drugs, and their use during pregnancy was analyzed.

### Outcome Variables

Maternal complications during pregnancy included pregnancy-induced hypertension, preeclampsia, hypoglycemia, and intrahepatic cholestasis. Data on maternal complications were obtained from the medical records according to the coding of diagnoses and information recorded by the treating clinicians. Therefore, maternal hypoglycemia was limited to episodes resulting in hospital admission.

Perinatal outcomes included gestational age at birth, preterm birth (before 37 weeks of gestation), the onset of labor (spontaneous), elective and emergency cesarean delivery, birthweight, congenital malformations, and neonatal complications (hypoglycemia and jaundice within 24 hours postpartum, and mortality within 7 days postpartum).

Gestational age was based on an ultrasound scan in the first trimester. Birthweight was standardized to infant sex and gestational age at birth and reported as a z-score using the Scandinavian standards of Marsal et al [17]. *Small for gestational age* (SGA) was defined as a standardized birthweight below the 10th percentile and *large for gestational age* (LGA) above the 90th percentile [18]. Congenital malformations diagnosed during pregnancy, or the postpartum hospital stay were registered using ICD-10. Regarding infant-related pregnancy outcomes, twin pregnancies contributed with 2 infants in the analyses.

### Statistical Analysis

Continuous data with a normal distribution were reported as means and SD, and data with a skewed distribution as medians and interquartile ranges. Visual inspection of Q-Q plots was used to determine the distribution of data. All categorical data were reported as counts and percentages. Group comparisons were performed using logistic and linear regression models with robust clustered standard errors to account for women contributing with multiple deliveries during the study period. A cluster analysis, taking the different hospitals into account, was omitted as the demography and treatment standards of the 4 hospitals were considered highly comparable. Continuous data with a skewed distribution were log-transformed before analyses.

The associations between potentially modifiable exposures (HbA1c per 10 mmol/mol increase, BMI per 1 kg/m^2^ increase, weight gain during pregnancy per 1 kg increase, and smoking) and adverse outcomes were examined by adjusted logistic regression models with robust clustered standard errors. In addition to the included modifiable exposure, we further adjusted for maternal age, ethnicity, and parity, as we deemed these to be potentially important confounders. We did not adjust the model for prepregnancy treatment with antihypertensives and statins, and presence of prepregnancy circulatory comorbidities, as these were considered part of the causal pathway. Results were reported as crude and adjusted odds ratios (aOR) with 95% CI and standard errors. Data were analyzed without imputation of missing data. A two-sided *P* value of <.05 was considered significant. Data analyses were performed using StataSE version 17.0 (StataCorp Ltd, College Station, Texas, USA).

## Results

### Study Population

**Figure 1.**
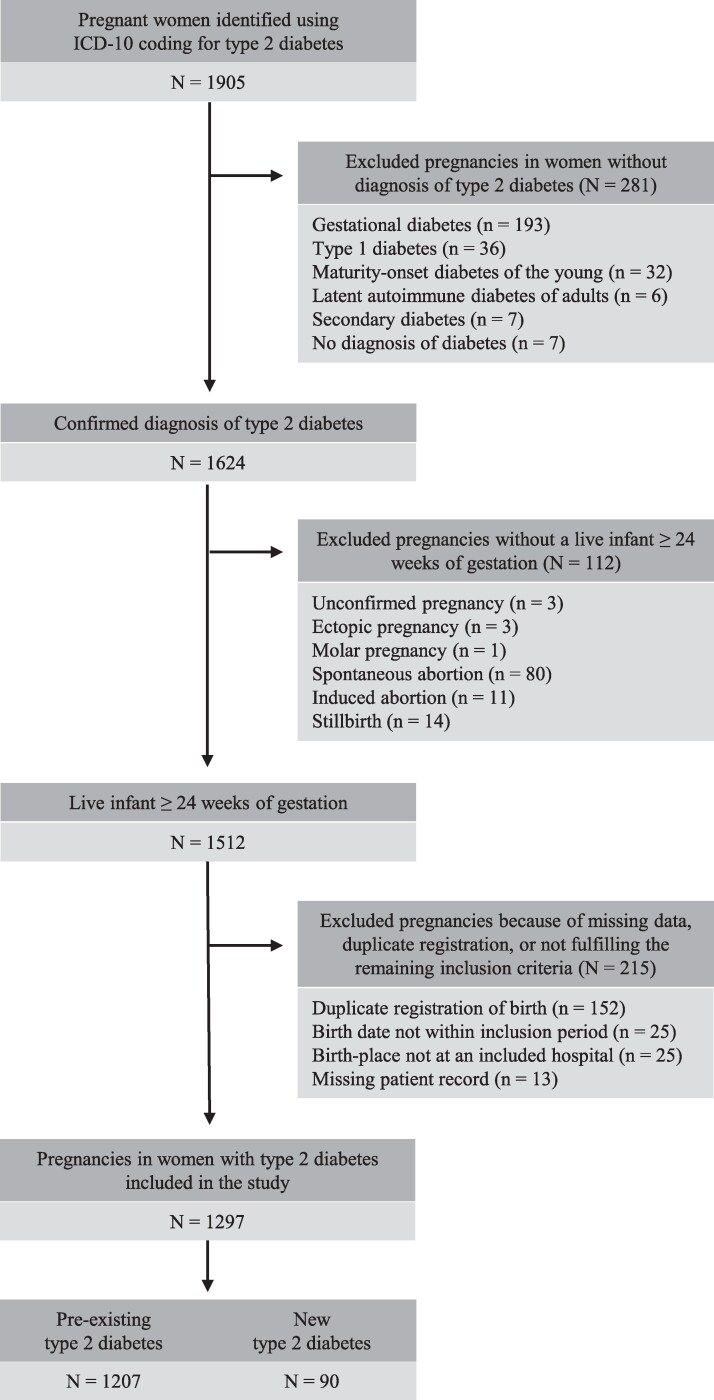
Flowchart of the study population. Inclusion criteria: Pregnant women with type 2 diabetes, giving birth to a live infant after 24 weeks of gestation at one of 4 University Hospitals in Denmark from January 1, 2004, until November 14, 2019. The study population was stratified into 2 groups: pregnancies in women with known preexisting type 2 diabetes (P-T2DM) and pregnancies in women with new type 2 diabetes first recognized during pregnancy and confirmed postpartum (N-T2DM). Abbreviation: ICD-10: ICD coding system 10th edition.

During the study period, 1905 pregnancies complicated by maternal T2DM were identified. After the screening of medical records, 608 pregnancies were excluded. Details of the excluded women are shown in Fig. 1. The final cohort consisted of 1297 pregnancies (93.1% P-T2DM, 6.9% N-T2DM). The number of women with T2DM giving birth increased almost 3-fold from 45 per year in 2004 to 122 per year in 2018.

### Population Characteristics

The total study population had a diverse ethnic background, including European (60.0%), Central-Western Asian (18.2%), South-East Asian (12.0%) and Afro-Caribbean (9.8%) (Table 1). At the time of delivery, the mean maternal age was 34.4 (5.4) years.

**Table 1.** Population characteristics in pregnant women with type 2 diabetes

	n	TOTAL n = 1297	n	P-T2DM n = 1207	n	N-T2DM n = 90	*P*
Maternal age, years*^a^*	1297	34.4 (5.4)	1207	34.5 (5.3)	90	34.2 (5.7)	.71
Nulliparous*^b^*	1295	398 (30.7)	1205	368 (30.5)	90	30 (33.3)	.58
Gestation at first visit, days*^c^*	1264	68 (35)	1176	67 (35)	88	76.5 (28.5)	<.001
Ethnicity*^b^*							.09
European	1286	772 (60.0)	1196	717 (60.0)	90	55 (61.1)	
Afro-Caribbean	1286	126 (9.8)	1196	118 (9.9)	90	8 (8.9)	
South-East Asian	1286	154 (12.0)	1196	137 (11.5)	90	17 (18.9)	
Central-Western Asian	1286	234 (18.2)	1196	224 (18.7)	90	10 (11.1)	
Smoking*^b^*	1277	261 (20.4)	1187	250 (21.1)	90	11 (12.2)	.050
Age at onset of diabetes, years*^a^*	1286	29.4 (6.2)	1197	29.0 (6.1)	89	33.7 (5.7)	<.001
Duration of diabetes, years*^c^*	1284	4.2 (4.8)	1196	4.5 (4.9)	—	—	—
Comorbidities*^b^*							
Somatic disorders	1296	476 (36.7)	1206	462 (38.3)	90	14 (15.6)	<.001
Psychiatric disorders	1296	157 (12.1)	1206	144 (12.0)	90	13 (14.4)	.48
Prepregnancy weight, kg*^a^*	1257	89.2 (21.0)	1168	89.0 (21.0)	89	91.4 (20.9)	.29
Prepregnancy BMI, kg/m^2^*^a^*	1246	32.6 (6.6)	1159	32.5 (6.7)	87	33.2 (6.1)	.33
Prepregnancy BMI, kg/m^2^*^b^*							<.001
Underweight (<18.5)	1246	7 (0.6)	1159	7 (0.6)	87	0 (0.0)	
Normal weight (18.5-24.9)	1246	151 (12.1)	1159	143 (12.3)	87	8 (9.2)	
Pre-obese (25.0-29.9)	1246	302 (24.2)	1159	285 (24.6)	87	17 (19.5)	
Obese (≥30.0)	1246	786 (63.1)	1159	724 (62.5)	87	62 (71.3)	
Weight change, kg*^a^*	1079	11.9 (7.6)	1009	12.1 (7.6)	70	9.0 (7.6)	.001
Hemoglobin A1c, mmol/mol*^c^*							
Prepregnancy	427	50 (15)	425	50 (15)	—	—	—
1st trimester	968	49 (15)	903	48 (14)	65	59 (22)	<.001
2nd trimester	1252	39 (8)	1164	39 (8)	88	40 (9)	.20
3rd trimester	1225	42 (10)	1139	42 (10)	86	43 (7)	.99
**Treatment prepregnancy**							
Systemic treatment*^b^*							
Antihypertensives	1291	171 (13.3)	1201	167 (13.9)	90	4 (4.4)	.017
Statins	1290	161 (12.5)	1200	159 (13.3)	90	2 (2.2)	.008
Antidepressants	1292	104 (8.1)	1202	96 (8.0)	90	8 (8.9)	.76
Teratogenic drug							
Treatment after conception*^b^*	1293	235 (18.2)	1203	231 (19.2)	90	4 (4.4)	.002
Discontinuation, days*^c^*	202	52 (38)	201	52 (38)	1	33 (0)	—
Treatment of diabetes*^b^*							
Insulin	1294	292 (22.6)	1204	292 (24.3)	—	—	—
Antidiabetic (not insulin)	1293	721 (55.8)	1203	716 (59.5)	—	—	—
No medicine	1294	397 (30.7)	1204	313 (26.0)	—	—	—
Insulin dose, IU/day*^c^*	280	40 (43)	280	40 (43)	—	—	—
**Treatment during pregnancy**							
Insulin treatment*^c^*							
1st trimester	1297	712 (54.9)	1207	675 (55.9)	90	37 (41.1)	.007
2nd trimester	1297	1143 (88.1)	1207	1055 (87.4)	90	88 (97.8)	.010
3rd trimester	1297	1189 (91.7)	1207	1101 (91.2)	90	88 (97.8)	.046
Max. insulin dose, IU/day*^c^*							
1st trimester	708	36 (42)	671	36 (44)	37	32 (22)	.008
2nd trimester	1127	60 (68)	1040	63 (72)	87	52 (38)	.012
3rd trimester	1177	92 (100)	1090	94 (104)	87	78 (70)	.074
Max. insulin dose, IU/kg/day*^c^*	1011	1.0 (0.9)	942	1.0 (0.9)	69	0.8 (0.7)	.13

Teratogenic drug: Treatment after conception, and days until discontinuation. Max. insulin dose: Maximum daily insulin dose in each trimester (IU/day), and in pregnancy (IU/day/kg). Statistical significance: A two-sided *P* value <.05.

Abbreviations: BMI, body mass index; N-T2DM, new type 2 diabetes; P-T2DM, preexisting type 2 diabetes.

^*a*^Mean (SD).

^*b*^Count (percentage).

^*c*^Median (interquartile range).

Cardiometabolic risk factors were prevalent before pregnancy. In the total study population, 20.4% were daily smokers, 13.3% were treated with antihypertensives, 12.5% with statins, and 63.1% had obesity (Table 1). Apart from chronic hypertension and dyslipidemia, the most common preexisting comorbidities were polycystic ovary syndrome (11.6%), thyroid disorders (8.0%), and affective disorders, primarily depression (8.6%) (Table 2).

**Table 2.** Maternal comorbidities in pregnant women with type 2 diabetes

	n	T2DM n = 1297	n	P-T2DM n = 1207	n	N-T2DM n = 90	*P*
Preexisting psychiatric disorder*^a^*							
Schizophrenia, schizotypal and delusional	1296	18 (1.4)	1206	17 (1.4)	90	1 (1.1)	.82
Affective	1296	112 (8.6)	1206	104 (8.6)	90	8 (8.9)	.93
Neurotic, stress-related and somatoform	1296	35 (2.7)	1206	33 (2.7)	90	2 (2.2)	.77
Personality	1296	8 (0.6)	1206	7 (0.6)	90	1 (1.1)	.54
Mental retardation	1296	2 (0.2)	1206	1 (0.1)	90	1 (1.1)	.017
Mental and behavioral	1296	3 (0.2)	1206	3 (0.3)	90	0 (0.0	—
Behavioral and emotional	1296	11 (0.9)	1206	9 (0.8)	90	2 (2.2)	.14
Preexisting somatic disorder*^a^*							
Endocrine and metabolic	1296	257 (19.8)	1206	251 (20.8)	90	6 (6.7)	.001
Thyroid gland disorders	1296	104 (8.0)	1206	100 (8.3)	90	4 (4.4)	.20
Ovarian dysfunction	1296	150 (11.6)	1206	148 (12.3)	90	2 (2.2)	.011
Circulatory system	1296	181 (14.0)	1206	178 (14.8)	90	3 (3.3)	.003
Chronic hypertension	1296	174 (13.4)	1206	171 (14.2)	90	3 (3.3)	.004
Respiratory system	1296	50 (3.9)	1206	48 (4.0)	90	2 (2.2)	.41
Digestive system	1296	19 (1.5)	1206	17 (1.4)	90	2 (2.2)	.54
Genitourinary system	1296	13 (1.0)	1206	12 (1.0)	90	1 (1.1)	.92
Musculoskeletal and connective tissue	1296	17 (1.3)	1206	17 (1.4)	90	0 (0.0)	—
Nervous system	1296	11 (0.9)	1206	10 (0.8)	90	1 (1.1)	.78
Blood and immune system	1296	11 (0.9)	1206	11 (0.9)	90	0 (0.0)	—
Neoplasms	1296	5 (0.4)	1206	4 (0.3)	90	1 (1.1)	.25
Bariatric surgery*^a^*	1295	31 (2.4)	1205	30 (2.5)	90	1 (1.1)	.43

Abbreviations: N-T2DM, new type 2 diabetes; P-T2DM, preexisting type 2 diabetes.

^*a*^Count (percentage). Statistical significance: A two-sided *P* value <.05.

Pregnant women with P-T2DM were more likely to be smoking and have somatic comorbidities compared to women with N-T2DM (Tables 1 and 2). Consequently, the prevalence of treatment with teratogenic drugs (statins, angiotensin-converting enzyme inhibitors, and angiotensin receptor blockers) was 19.2% at the time of conception in women with P-T2DM. The median number of days from conception until discontinuation was 52 [19] days, resulting in fetal exposure during most of the first trimester (Table 1). Only 4.4% of women with N-T2DM were treated with teratogenic drugs after conception.

### Weight Gain

**Figure 2.**
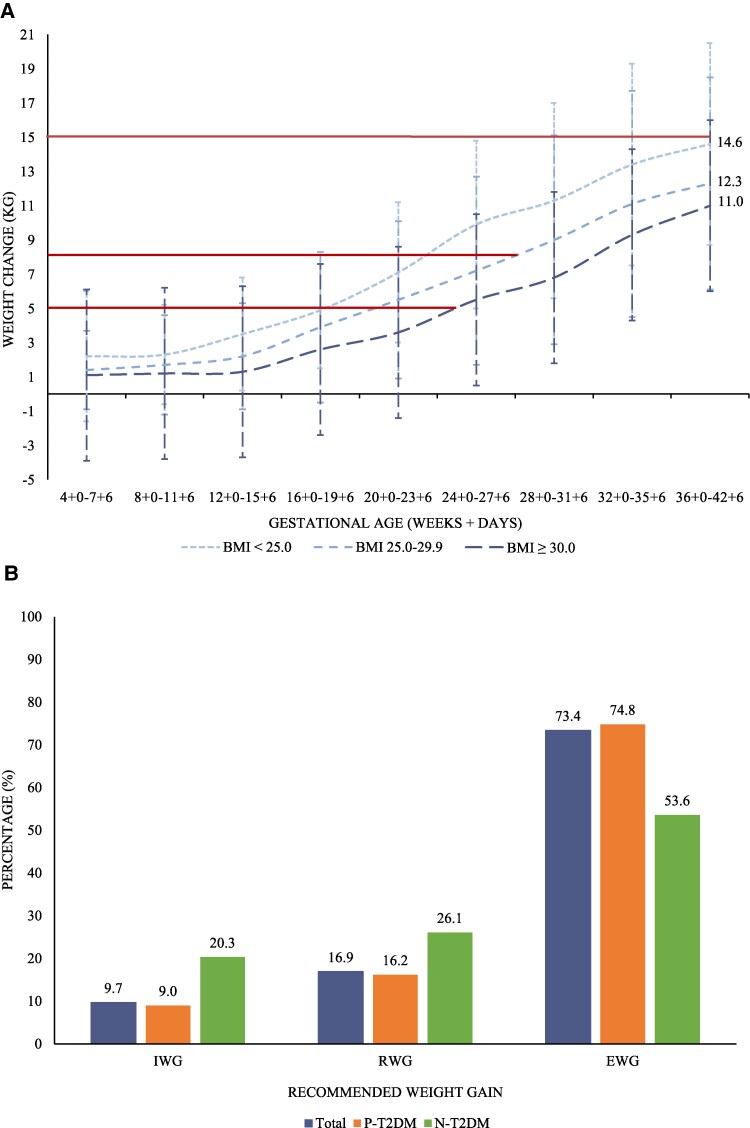
Weight change during pregnancy in women with type 2 diabetes. A, Mean weight change (kg) during pregnancy in the total study population stratified by prepregnancy BMI. B, Percentage (%) distribution of women according to recommended weight gain during pregnancy. Error bars represent SD. Horizontal red lines represent the maximum recommended weight gain during pregnancy in women with preexisting diabetes stratified by BMI according to Danish guidelines. Abbreviations: EWG, excessive weight gain; IWG, inadequate weight gain; N-T2DM, new type 2 diabetes (green); P-T2DM, preexisting type 2 diabetes (orange); RWG, recommended weight gain.

In the total study population, mean weight gain during pregnancy was 11.9 (7.6) kg (Table 1). Examining weight gain according to BMI, only women with a BMI <25.0 kg/m^2^ attained the recommended mean weight gain (Fig. 2A). Most notably, women with a BMI ≥30.0 kg/m^2^ had a mean weight gain of 11.0 (7.9) kg, more than twice the recommended amount. In total, 73.4% of women had an excessive weight gain (Fig. 2B). Women with P-T2DM gained more weight than N-T2DM.

### Glycemic Control

**Figure 3.**
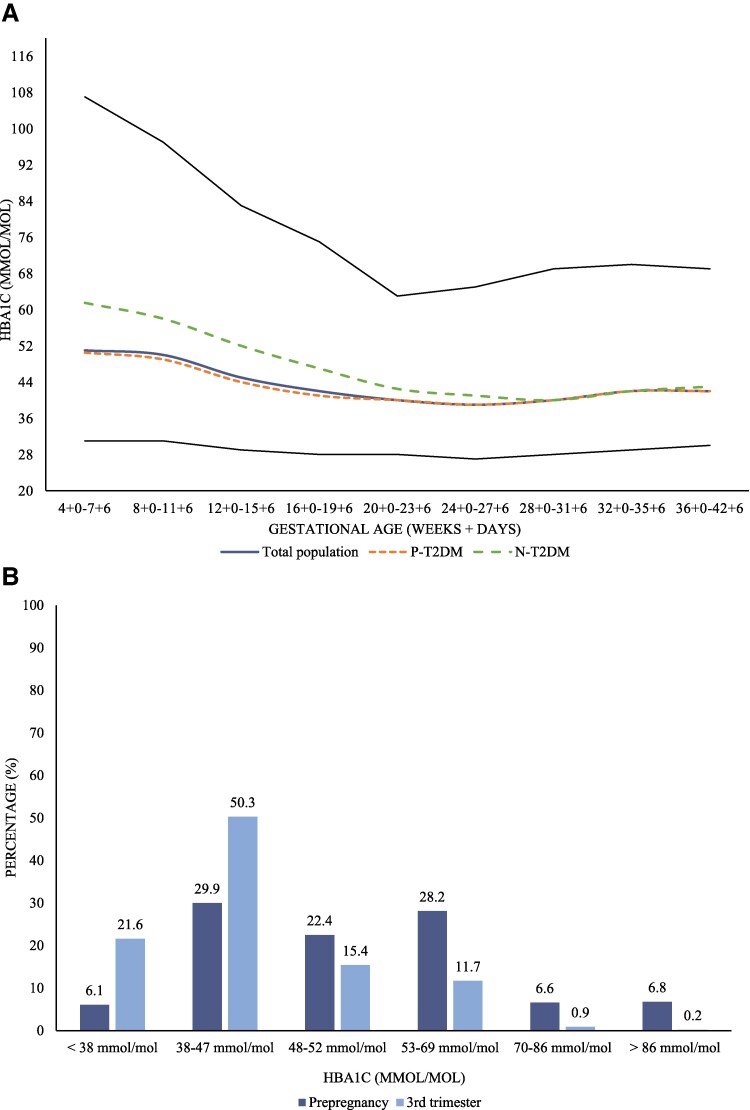
Glycemic control during pregnancy in women with type 2 diabetes. A, Median HbA1c (mmol/mol) during pregnancy. B, Percentage (%) distribution of women according to prepregnancy HbA1c and HbA1c in the third trimester in the total study population. Black lines represent the 99th and 1st percentiles. Abbreviations: P-T2DM, preexisting type 2 diabetes (orange); N-T2DM, new type 2 diabetes (green).

In the total study population, median HbA1c seemed to decrease until mid-pregnancy (Fig. 3A). However, median HbA1c in the third trimester was still 42 (10) mmol/mol (6.0%) (Table 1). Prepregnancy HbA1c measured less than 3 months prior to pregnancy was only available for 35.2% of pregnancies complicated by P-T2DM. Among those with an available HbA1c, only 58.6% had a prepregnancy HbA1c <53 mmol/mol (7.0%) as recommended (Fig. 3B). Only 21.6% attained a median HbA1c <38 mmol/mol (5.6%) (Fig. 3B). Women with P-T2DM had a lower median HbA1c in the first trimester compared to N-T2DM, but the groups attained a similar HbA1c during the second trimester (Table 1).

### Insulin Treatment

**Figure 4.**
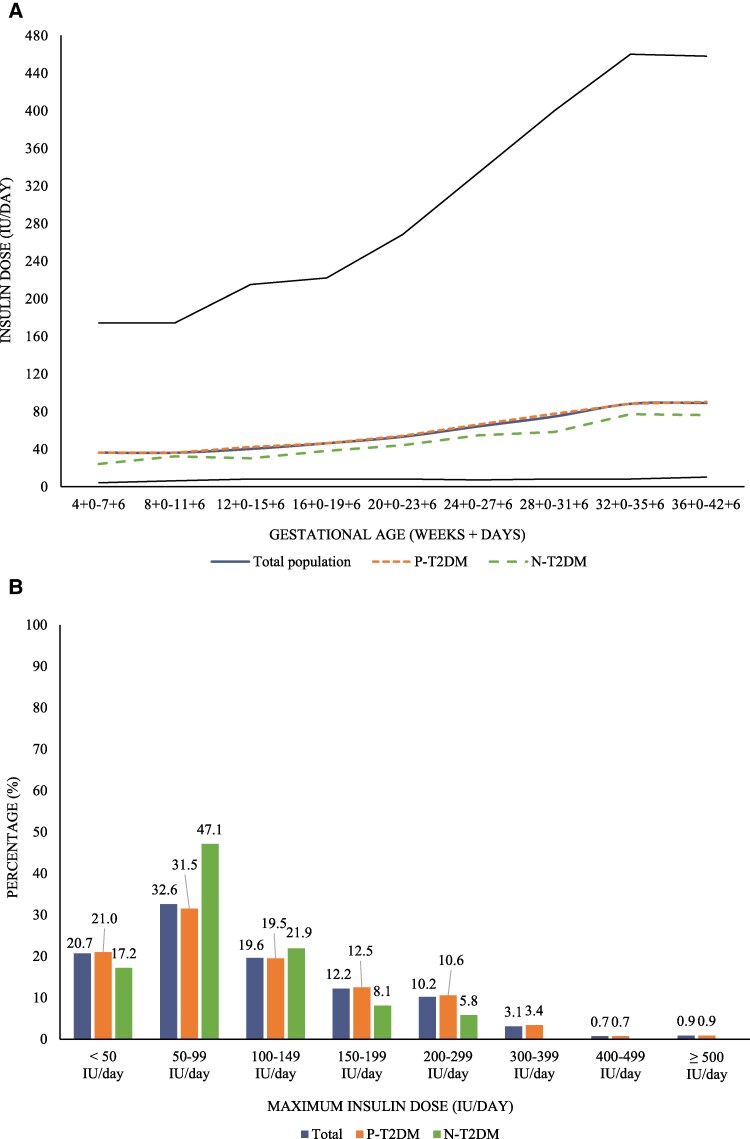
Daily insulin dose during pregnancy in women with type 2 diabetes. A. Median daily insulin dose (IU/day) during pregnancy. B. Percentage (%) distribution of women according to maximum daily insulin dose (IU/day) in the 3rd trimester. Black lines represent the 99th and 1st percentiles. Abbreviations: N-T2DM, new type 2 diabetes (green); P-T2DM, preexisting type 2 diabetes (orange).

Most women needed insulin treatment during pregnancy (91.7%) (Table 1). In the total study population, the median daily insulin dose seemed to increase from 36 (32) IU/day in early pregnancy to 89 (59) IU/day in late pregnancy (Fig. 4A). The maximum daily insulin dose during pregnancy was ≥100 IU/day in 46.7%, and ≥200 IU/day in 14.9% (Fig. 4B). Women with P-T2DM started with a higher daily insulin dose, but there was no difference between the groups in the third trimester (Table 1).

### Obstetric and Perinatal Outcomes

During pregnancy, 15.4% were diagnosed with pregnancy-induced hypertension, and in late pregnancy, preeclampsia was present in 9.1% of the total study population (Table 3). Few women had a spontaneous onset of labor (6.9%), and emergency cesarean delivery was common (26.6%). The median length of the pregnancy was 37 weeks and 6 days, with 19.0% delivering preterm. The prevalence of neonatal deaths was 0.1%, and 5.0% of infants were diagnosed with a congenital malformation. The standardized birthweight z-score was 0.65 (1.69), with 32.2% of infants being LGA and 11.4% SGA. Women with P-T2DM were less likely to have a spontaneous onset of labor compared to women with N-T2DM (6.1% vs 16.7%, *P* < .001) and potentially more likely to have an emergency cesarean delivery (27.4% vs 16.7%, *P* .090) (Table 3). All other outcomes were similar in the 2 groups.

**Table 3.** Obstetric and perinatal outcomes in women with type 2 diabetes

	n	T2DM n = 1297	n	P-T2DM n = 1207	n	N-T2DM n = 90	*P*
Maternal complications*^a^*							
Severe hypoglycemia	1292	15 (1.2)	1202	15 (1.3)	90	0 (0.0)	—
Hypertension	1296	200 (15.4)	1206	181 (15.0)	90	19 (21.1)	.13
Preeclampsia	1297	118 (9.1)	1207	109 (9.0)	90	9 (10.0)	.76
Intrahepatic cholestasis	1296	18 (1.4)	1206	16 (1.3)	90	2 (2.2)	.46
Infants per pregnancy*^a^*							
Singleton pregnancy	1297	1265 (97.5)	1207	1175 (97.4)	90	90 (100.0)	—
Twin pregnancy	1297	32 (2.5)	1207	32 (2.7)	90	0 (0.0)	—
Infant sex, girl*^a^*	1327	650 (49.0)	1237	609 (49.2)	90	41 (45.6)	.50
Gestation at birth, days*^b^*	1297	265 (10)	1207	265 (9)	90	266 (11)	.44
Preterm birth < 37 weeks*^a^*	1297	246 (19.0)	1207	231 (19.1)	90	15 (16.7)	.56
Onset of labor, spontaneous*^a^*	1295	89 (6.9)	1205	74 (6.1)	90	15 (16.7)	<.001
Mode of delivery*^a^*							.090
Vaginally	1295	622 (48.0)	1205	574 (47.6)	90	48 (53.3)	
Elective cesarean delivery	1295	328 (25.3)	1205	301 (25.0)	90	27 (30.0)	
Emergency cesarean delivery	1295	345 (26.6)	1205	330 (27.4)	90	15 (16.7)	
Apgar score 10 minutes, <7 points*^a^*	1099	4 (0.4)	1026	3 (0.3)	73	1 (1.4)	.18
Birthweight, g*^c^*	1311	3342 (729)	1224	3334 (738)	87	3465 (585)	.050
Birthweight z-score*^c^*	1311	0.65 (1.69)	1224	0.65 (1.70)	87	0.77 (1.58)	.48
Birthweight*^a^*							.58
SGA	1311	150 (11.4)	1224	142 (11.6)	87	8 (9.2)	
AGA	1311	739 (56.4)	1224	692 (56.5)	87	47 (54.0)	
LGA	1311	422 (32.2)	1224	390 (31.9)	87	32 (36.8)	
Head circumference, cm*^c^*	1153	34.5 (2.1)	1071	34.5 (2.1)	82	34.7 (1.9)	.23
Abdominal circumference, cm*^c^*	926	32.7 (2.7)	863	32.7 (2.7)	63	33.0 (2.1)	.22
Length, cm*^c^*	1237	50.5 (3.3)	1153	50.5 (3.3)	84	50.6 (2.7)	.77
Placental weight, g*^c^*	946	716 (194)	881	718 (196)	65	696 (173)	.35
Congenital malformation*^a^*	1311	66 (5.0)	1222	61 (5.0)	89	5 (5.6)	.80
Congenital malformation*^d^*	1311	50.3	1222	49.9	89	56.2	—
Congenital malformation*^a^*							
Nervous system	1311	3 (0.2)	1222	3 (0.3)	89	0 (0.0)	—
Circulatory system	1311	27 (2.1)	1222	25 (2.1)	89	2 (2.3)	—
Respiratory system	1311	2 (0.2)	1222	2 (0.2)	89	0 (0.0)	—
Cleft lip and palate	1311	10 (0.8)	1222	9 (0.7)	89	1 (1.1)	—
Digestive system	1311	0 (0.0)	1222	0 (0.0)	89	0 (0.0)	—
Genital organs	1311	3 (0.2)	1222	3 (0.3)	89	0 (0.0)	—
Urinary system	1311	7 (0.5)	1222	7 (0.6)	89	0 (0.0)	—
Musculoskeletal	1311	4 (0.3)	1222	4 (0.3)	89	0 (0.0)	—
Chromosomal	1311	3 (0.2)	1222	3 (0.3)	89	0 (0.0)	—
Multiple anomalies	1311	7 (0.5)	1222	5 (0.4)	89	2 (2.3)	—
Neonatal complications*^a^*							
Hypoglycemia	1037	209 (20.2)	964	191 (19.8)	73	18 (24.7)	.32
Jaundice	1039	26 (2.5)	966	25 (2.6)	73	1 (1.4)	.53
Mortality	1050	1 (0.1)	976	0 (0.0)	74	1 (1.4)	—

Maternal complications: Hypoglycemia is limited to events resulting in hospital admittance, and hypertension is limited to pregnancy-induced (not preexisting). Birthweight z-score: Adjusted for infant sex and gestational age at birth. Neonatal complications: Hypoglycemia ≤24 hours postpartum, jaundice ≤24 hours postpartum, and mortality ≤7 days postpartum. Statistical significance: A two-sided *P* value <.05 when comparing P-T2DM and N-T2DM.

Abbreviations: AGA, appropriate for gestational age; LGA, large for gestational age; N-T2DM, new type 2 diabetes; P-T2DM, preexisting type 2 diabetes; SGA, small for gestational age.

^*a*^Count (percentage).

^*b*^Median (interquartile range).

^*c*^Mean (SD).

^*d*^Rate per 1000 births.

Using logistic regression models, third-trimester HbA1c (aOR 2.49, 95% CI 1.70-3.63), prepregnancy BMI (aOR 1.05, 95% CI 1.02-1.08), weight gain during pregnancy (aOR 1.06, 95% CI 1.03-1.09), and smoking (aOR 0.59, 95% CI 0.38-0.92) were all independently associated with LGA birthweight (Table 4). HbA1c in the first trimester (aOR 1.79, 95% CI 1.34-2.37) was associated with congenital malformation. Finally, prepregnancy BMI and weight gain during pregnancy were both associated with preeclampsia and cesarean delivery, and prepregnancy BMI and second trimester HbA1c with preterm birth (Table 4). Notably, prepregnancy HbA1c was not included in the logistic regression model, as it was missing for most women with P-T2DM and unavailable for women with N-T2DM.

**Table 4.** Adjusted multiple logistic regression analysis for adverse obstetric and perinatal outcomes in singleton pregnancies

	Preeclampsia n = 107			Preterm birth n = 225			Cesarean delivery n = 647		
	aOR	95% CI	SE	aOR	95% CI	SE	aOR	95% CI	SE
1st trimester HbA1c, mmol/mol	1.10	0.87 1.39	0.13	0.97	0.81 1.17	0.09	1.06	0.92 1.23	0.08
2nd trimester HbA1c, mmol/mol	1.56	0.83 2.93	0.50	**1.95**	1.23 3.09	0.46	1.21	0.83 1.76	0.23
3rd trimester HbA1c, mmol/mol	0.82	0.43 1.56	0.02	0.94	0.62 1.42	0.20	1.11	0.78 1.58	0.20
Prepregnancy BMI, kg/m^2^	**1**.**06**	1.02 1.11	0.02	**1**.**04**	1.01 1.08	0.02	**1**.**03**	1.01 1.06	0.01
Weight gain during pregnancy, kg	**1**.**09**	1.04 1.13	0.02	1.03	0.99 1.06	0.02	**1**.**03**	1.00 1.05	0.01
Smoking (Ref. No)	0.80	0.41 1.54	0.27	1.12	0.67 1.90	0.30	1.06	0.71 1.60	0.22

	Small for gestational age n = 123			Large for gestational age n = 421			Congenital malformation n = 64		
	aOR	95%CI	SE	aOR	95%CI	SE	aOR	95%CI	SE
1st trimester HbA1c, mmol/mol	1.09	0.85 1.40	0.14	0.88	0.75 1.03	0.07	**1**.**64**	1.29 2.08	0.20
2nd trimester HbA1c, mmol/mol	1.52	0.66 3.49	0.65	1.00	0.67 1.50	0.21	—	—	—
3rd trimester HbA1c, mmol/mol	**0**.**34**	0.16 0.70	0.12	**2**.**49**	1.70 3.63	0.48	—	—	—
Prepregnancy BMI, kg/m^2^	**0**.**94**	0.90 0.99	0.02	**1**.**05**	1.02 1.08	0.02	1.04	0.99 1.09	0.03
Weight gain during pregnancy, kg	0.98	0.92 1.04	0.03	**1**.**06**	1.03 1.09	0.02	0.98	0.92 1.03	0.03
Smoking (ref. No)	1.52	0.83 2.77	0.47	**0**.**59**	0.38 0.92	0.13	2.09	0.91 4.77	0.88

Total number of pregnancies included in the analysis n = 725. Adjusted for maternal age (years), ethnicity (group), and parity (number). HbA1c increase with 10 mmol/mol per unit. Prepregnancy BMI and weight gain during pregnancy increases with 1 kg/m^2^ and 1 kg per unit, respectively. HbA1c was defined as the median of all HbA1c values measured within the trimester. The 2nd and 3rd trimester HbA1c was omitted from congenital malformations due to the organogenesis occurring in the 1st trimester. Prepregnancy HbA1c was omitted, as it reduced the sample size significantly. Preterm birth: Before 37 weeks of gestation. Small for gestational age: Standardized birthweight <10th percentile. Large for gestational age: Standardized birthweight >90th percentile. Cesarean delivery: Emergency and elective. Congenital malformation: Major and minor malformations. Bold indicates a statistically significant OR.

Abbreviations: BMI, body mass index; HbA1c, hemoglobin A1c; N-T2DM, new type 2 diabetes; OR, odds ratio; P-T2DM, preexisting type 2 diabetes; SE, standard error (clustered).

## Discussion

This nationwide study revealed that preexisting cardiometabolic risk factors were prevalent in pregnant women with T2DM, including smoking, hypertension, dyslipidemia, obesity, and hyperglycemia. In addition, most had excessive weight gain during pregnancy, only 21.6% attained an HbA1c <38 mmol/mol (5.6%) in the third trimester, and 46.7% needed high-dose insulin treatment (more than 100 IU/day) in late pregnancy. Modifiable cardiometabolic risk factors (HbA1c during pregnancy, prepregnancy BMI, weight gain during pregnancy, and smoking) were all associated with one or more severe adverse outcomes during pregnancy.

As expected, HbA1c was higher, and the need for insulin treatment and doses were lower at the beginning of pregnancy in women with N-T2DM, who likely had unrecognized and untreated diabetes. The main differences between the 2 groups were the higher prevalence of preexisting comorbidities and smoking in women with P-T2DM. However, as T2DM was diagnosed during pregnancy in women with N-T2DM, concomitant diseases may also have been unrecognized before pregnancy. Adverse obstetric and perinatal outcomes were prevalent in the study population, and unexpectedly, outcomes were similar in the 2 groups. An explanation for this could be that the women with P-T2DM had rather well-controlled diabetes prior to pregnancy and the majority were not treated with insulin prior to pregnancy, indicating relatively mild diabetes.

To our knowledge, only 2 previous studies have described the prevalence of risk factors and adverse outcomes in pregnant women with T2DM in a study population of this size [6, 12]. Furthermore, this is the largest study on pregnant women with T2DM describing longitudinal changes in HbA1c, weight gain, and daily insulin dose across gestation [7-10, 20].

Murphy et al recently published a cohort study from the United Kingdom, including 8684 pregnant women with T2DM, aiming to identify risk factors associated with adverse pregnancy outcomes [6]. As in the current study, one of the most important risk factors in pregnancy was elevated HbA1c. However, Murphy et al did not include data on weight gain during pregnancy. In our study, excess weight gain was associated with multiple adverse outcomes in the current study, including LGA.

Notably, Murphy et al used the National Institute for Health and Care Excellence target of HbA1c <48 mmol/mol (6.5%) during pregnancy, which differs from the recommendations in Denmark. Applying the same recommendations to the current study population, 36.1% had an HbA1c <48 mmol/mol (6.5%) before conception, and 71.9% attained the target HbA1c in the third trimester, similar to the results reported by Murphy et al [6]. It is concerning that so many women present with an elevated HbA1c at conception and struggle to attain target HbA1c during pregnancy.

In pregnancy, HbA1c is generally used as an adjunct to capillary blood glucose measurements for glycemic monitoring [14]. However, HbA1c in itself is an important prognostic biomarker. Elevated periconceptional HbA1c is known to be associated with higher odds of having an infant with congenital malformation(s) [6, 21-23], and elevated third trimester HbA1c is associated with higher odds of LGA birthweight [6, 24] and perinatal death [6, 25]. In women with T1DM, elevated HbA1c has also been associated with preeclampsia and preterm delivery, but results have been conflicting in women with T2DM [6, 26, 27]. The current study found no association between HbA1c and preeclampsia, but preterm birth was associated with increased HbA1c in the 2nd trimester. Therefore, appropriate glycemic control is essential.

Current guidelines for pregnant women with preexisting diabetes are primarily based on studies on women with T1DM, and recommended glycemic targets may not be immediately transferable to women with T2DM [14, 22]. HbA1c is generally lower in pregnant women with T2DM compared to pregnant women with T1DM [6-11, 13, 20], and a study from Ireland suggests that the HbA1c level at which adverse outcomes occur may also be lower [10].

In the current study, HbA1c decreased initially but stabilized from mid-pregnancy. Previous studies in T2DM pregnancy from Australia, Ireland and Denmark found similar changes [9, 13, 20]. A Danish study on longitudinal HbA1c measurements in pregnant women with T1DM concluded that HbA1c levels remained stable after gestational weeks 19 to 22 [28], and that women with high prepregnancy HbA1c did not attain target HbA1c during pregnancy [28]. These findings indicate that improving glycemic control sufficiently during the limited timeframe from conception until delivery may be challenging, emphasizing the importance of optimizing prepregnancy glycemic control.

The course of HbA1c during pregnancy has recently been described in detail by our group [29]. This paper was based on a subgroup of the women in the current study and identified and characterized HbA1c trajectories across gestation. Three HbA1c trajectory groups were identified and named according to the glycemic control in early pregnancy. Women with poor glycemic control in early pregnancy had lower odds of having an infant with LGA, and higher odds of having an infant with SGA and congenital malformations compared with women with good glycemic control. There was no evidence of a difference in odds of preeclampsia, preterm birth, and cesarean delivery between groups.

This is, to some extent, in line with the current study as HbA1c in the third trimester was independently associated with LGA birthweight and HbA1c in the first trimester was associated with congenital malformations. However, second trimester HbA1c was associated with preterm birth in the current study. The 2 studies are somewhat different in the statistical methods approach and study sample, which could explain why the conclusions are slightly different, albeit pointing in the same direction.

Previous studies have found a high risk of adverse obstetric and perinatal outcomes associated with maternal T2DM comparable to maternal T1DM [6, 7, 11, 20], confirming the severity of the disease. Murphy et al concluded that the prevalence of congenital malformations and stillbirths was similar. In contrast, the prevalence of LGA infants and preterm deliveries was higher in women with T1DM, and the prevalence of neonatal deaths and SGA infants was higher in women with T2DM [6]. In the current study, the prevalence of congenital malformations was 5.0%, equivalent to a rate of 50.3 per 1000 births. Murphy et al found a slightly lower prevalence of congenital malformations at 4.5% (40.5 per 1000 births) [6]. Both studies only included malformations diagnosed antenatally or during the postpartum hospital stay. Without longer follow-up, the risk of malformations may be underestimated. However, a Danish register-based study found a prevalence of congenital malformations at 5.2% (95% CI 3.3%-7.2%) in children born to mothers with T2DM, equivalent to a rate of 52.2 per 1000 births, with the highest risk found to be cardiovascular malformations [22]. As this study used register-based data from 2000 to 2017 with a minimum follow-up time of 2 years after birth, we hypothesize that the potential number of missed malformations in the current study would not alter the results significantly.

The most prevalent adverse pregnancy outcome in the current study was having an LGA infant. This was independently associated with increased third trimester HbA1c, BMI, and weight gain during pregnancy, in agreement with previous studies [6, 30, 31]. In other recent studies, the risk of having an LGA infant has been reported between 20.0% and 38.4% [7, 8, 10, 12]. This is concerning, as having an LGA infant has been associated with an increased risk of prolonged labor, cesarean delivery, shoulder dystocia, and brachial plexus trauma [1], in addition to long-term childhood morbidities such as obesity, diabetes, and cardiovascular disease [32].

Initiating glucose-lowering treatments to prevent hyperglycemia can improve pregnancy outcomes [1]. However, insulin needs were found to be very heterogeneous in the current study, which may complicate the care for these women and result in inadequate doses in women with rapidly changing needs. As the pregnancies progressed, we observed that the variation in daily insulin dose increased, with women receiving between 10 and 500 IU/day before delivery. This variation may be explained by body weight and biological differences. Three other small studies examined the daily insulin dose in pregnant women with T2DM [8, 13, 33]. A study from Australia reported a rapid increase in insulin dose throughout pregnancy [13], similar to the current study. A study from the United States found that 45.0% of pregnant women with T2DM needed high-dose insulin treatment (defined as ≥2 IU/kg/day) [12]. Factors that may influence the severity of insulin resistance are obesity, excessive weight gain, and polycystic ovary syndrome [34, 35], all prominent in the current study population.

To improve pregnancy outcomes, the first step could be to tailor a prepregnancy care program to women with T2DM [36]. A few decades ago, it would have been rare to diagnose T2DM in women below 45 years, but the prevalence of early-onset T2DM is increasing worldwide [2, 37]. Compared to people with late-onset T2DM, people with early-onset have more obesity and are less physically active, have poorer glycemic control and have a higher prevalence of hypertension, dyslipidemia, albuminuria, retinopathy, and smoking at the time of diagnosis [19, 38, 39]. This is consistent with the results obtained in the current study, emphasizing severe multimorbidity in women with T2DM in the reproductive age planning pregnancy.

Another consideration is the association between affective disorders and T2DM [40]. In the current study, 8.6% of women were diagnosed with affective disorders. Therefore, the care of pregnant women with T2DM may benefit from offering psychological support. It may also be important to consider the ethnic diversity of the population, as there could be language barriers and cultural differences impacting the understanding and interpretation of healthcare recommendations.

We present data from a large multi-ethnic national cohort with an extensive list of variables creating a multifaceted characterization of pregnancies in women with T2DM. Another major strength of the study is the inclusion of data unavailable through national registries and previously not described in detail in pregnant women with T2DM in a study population of this size. The results presented here may have implications for future prepregnancy counseling and antenatal care in similar populations.

A limitation of the study is that it is based on data from a Danish cohort and although the cohort is multi-ethnic it may not represent women with T2DM in other regions. Another limitation is that our analyses did not adjust for multiple comparisons, which could potentially increase the risk of false positives. However, our logistic regression results revealed consistent associations between weight and glycemic parameters and adverse maternal and perinatal outcomes that were also physiologically plausible. Moreover, the exclusion of stillbirths, defined as fetal death after 24 weeks of gestation is a limitation as it could have affected the number of congenital malformations. In addition, there was one case of neonatal death, limiting our statistical power on this critical but rare outcome. Moreover, it must be kept in mind that the number of women with N-T2DM was small, and thus a potential difference could be difficult to demonstrate when comparing the small group of women with N-T2DM with the much larger group of women with P-T2DM.

Cohort studies are sensitive to selection bias, and this risk is considerable in the current study. First, the identification of the cohort relied on the referral of all pregnant women with T2DM to one of the inclusion hospitals, and ICD-10 code being assigned to all relevant pregnancies. Therefore, there is a possibility that some eligible pregnancies have been missed during our search. Second, some women fulfilling the criteria for N-T2DM may have been undiagnosed during the pregnancy. We believe that it is unlikely, that most women with unrecognized T2DM would not develop glucosuria during pregnancy, as the insulin sensitivity decreases drastically. However, it is possible that women with a mild degree of T2DM would remain undiagnosed during pregnancy. Lastly, a limitation of a retrospective cohort study is the risk of bias due to incomplete adjustment of the examined associations. Residual confounding could have been caused by socioeconomic factors, which were not available in the current study. Furthermore, the current study did not include data on the presence of microvascular complications of diabetes. However, data on known risk factors for the development of microvascular complications such as duration of diabetes, hyperglycemia, chronic hypertension, dyslipidemia, and smoking were reported.

## Conclusions

The prevalence of T2DM in pregnancy is increasing rapidly worldwide, but most guidelines for diabetes during pregnancy are based on studies in women with T1DM. As a result, there is an urgent need to improve knowledge regarding pregnant women with T2DM. This nationwide study revealed that preexisting cardiometabolic risk factors are prevalent in pregnant women with T2DM, including smoking, hypertension, dyslipidemia, obesity, and dysregulated glycemia and are associated with severe adverse outcomes for both mother and child. Women with T2DM diagnosed before pregnancy were found to have better glycemic control in early pregnancy compared to women with T2DM diagnosed during pregnancy, but the prevalence of adverse outcomes was similar. Interventions targeting the multimorbidity and treatment challenges faced by pregnant women with T2DM both before and during pregnancy should be prioritized.

## Data Availability

The datasets generated and analyzed during the current study are not publicly available due to the General Data Protection Regulation in Denmark. Still, they are available from the corresponding author on reasonable request and with permission of the Regional Council in the Central Denmark Region.

## Abbreviations

aOR: adjusted odds ratio
BMI: body mass index
HbA1c: glycated hemoglobin
ICD: International Classification of Diseases
LGA: large for gestational age
N-T2DM: new type 2 diabetes mellitus
P-T2DM: preexisting type 2 diabetes mellitus
SGA: small for gestational age
T1DM: type 1 diabetes mellitus
T2DM: type 2 diabetes mellitus
